# 

**Published:** 1850-08

**Authors:** 


### NOTICE TO CORRESPONDENTS.

Communications and Books for notice should be addressed to the Editor, care of Messrs. Lindsay & Blakiston.

Letters, &c., connected with the *business affairs* of the Journal should be addressed to the Publishers.

Papers for publication must be received *before* the 20th of the month, or they cannot appear in the forthcoming number.

The following Journals have been received in exchange:

The Boston Medical and Surgical Journal. (Weekly, Boston.)
Buffalo Journal.
Medical News.
Western Lancet.
Western Joural.
North-Western Medical and Surgical Journal.
The British American Journal of Medical and Physical Science. (Montreal.)
The London Lancet. (Weekly, London.)
The Medical Times. (Weekly, London.)
Dublin Medical Press.
Provincial Medical and Surgical Journal.

---

The following works have also been received for notice:

Essays on the Puerperal Fever, by F. Churchill, M. D. From Messrs. Lea & Blanchard.

Taylor's Medical Jurisprudence. From Messrs. Lea & Blanchard.

Essay on Alcoholic Drinks; by Wm. B. Carpenter, M. D. From the same.

Experiments on Warming and Ventilating Hospitals. By T. S. Kirkbride, M. D.

Proceedings of the Medical Society of North Carolina.

Transactions of the Medical Society of the State of New York.

Historical Sketch of the state of Medicine in the American Colonies. By J. B. Beck, M. D.

Annual Announcement of Jefferson Medical College.

Maclise's Surgical Anatomy, No. 3.

Life and correspondence of Dr. Andrew Combe.

Address to the Graduates of Baltimore College of Dental Surgery. By E. Townsend, D. D. S.

Annual Announcement of Baltimore College of Dental Surgery. Session of 1850–1.

Valedictory Address of Baltimore College of Dental Surgery. By S. P. Hulehen, M. D., D. D. S.

Dunglison's Materia Medica, Fourth Edition. Lea & Blanchard.

---

Communications have been received from:—

W. J. Reese, M. D., Alabama.
William Waters, M. D., Fredericktown, Md.
C. P. Johnson, M. D., Richmond, Va.
J. Travis, M. D., Mansfield, Tenn.
O. H. Edwards, Clinton, Summit Co., Ohio.
G. H. H. Koontz, Woodstock, Va.
E. C. Banks, Lawrenceville, Ill.

---

The foreign correspondents of the Examiner will please direct their Exchanges and other communications to the care of Mr. Charles J. Skeet, 27 King William St., Charing Cross, London, or Mr. H. Bossange, 21 Bis, Quai Voltaire, Paris.

